# A Doctor’s Story

**Published:** 1910-06

**Authors:** 


### Abstracts and Selections.

### A Doctor's Story.

"The Strange Case of Dr. Bruno." * * * The case is ingeniously invented. A gentleman of wealth, and German descent and education, who has his happiness wrecked by a conspiracy of cruel fate, goes to school to the mud wasp or "dirt-dauber" to find a drug which will suspend all the faculties in such a fashion as to preserve life under the appearance of death. Thus the mud wasp preserves the spider as fresh meat for her young, and Dr. Bruno obtains a fluid chemically similar to that in the sac behind the wasp's sting, which he tries upon mice, pigs, and monkeys till he is sure enough of his results to experiment with himself.

He times the dose for six months and one hour precisely, and lies down to pass that space in a state of animation so suspended that, as there shall be little or no waste, so there shall be no need of sustenance. The outcome of the experiment appears in the story, which deals very tragically with the passion of love and involves two beautiful women and two very handsome men. The scene is laid in New Orleans and in the city of Jackson, Miss., and the author, F. E. Daniel, M. D., has availed himself of all the tricks of melodrama to spice the dish of his medical fancy. The medical fancies are better than the rest—have, in fact, a quite convincing air of verisimilitude.—*From Times Book Review.*

A few years ago the New Orleans *Picayune* published a remarkable story of a man who was lying in a condition closely resembling death in the house and under the care of an eminent oculist and an equally eminent physician, both of New Orleans. Some months after, the paper again referred to the case, the subject having then been in a comatose state for nearly six months. By this time the facts had become known to the leading physicians of the city, who were much puzzled and were watching for the outcome with great interest. The case was discussed then and is still referred to as "The Strange Case of Dr. Bruno."

The oculist has since died, leaving among his manuscripts a full account of the affair, and his heir and executor, Dr. Ferdinand E. Daniel, has given it to the world. A stranger story was never penned.

Dr. Bruno, who, after years of study, tried the experiment upon himself of suspending animation without destroying life, was a fellow student of the oculist at Heidelberg, and they became intimate friends. He was quite successful in the experiment, and lay in a comatose state, without food or water, for six months. * * * He had left his story in manuscript. Aside from the experiment, Dr. Bruno's life was curious and eventful, containing a romance which the reader will eagerly follow. Dr. Daniel was President of the American International Congress of Physicians which closed its sessions at the Hotel Astor last week.—*From New York Commercial.*

